# The role of acetyl xylan esterase in the solubilization of xylan and enzymatic hydrolysis of wheat straw and giant reed

**DOI:** 10.1186/1754-6834-4-60

**Published:** 2011-12-20

**Authors:** Junhua Zhang, Matti Siika-aho, Maija Tenkanen, Liisa Viikari

**Affiliations:** 1 College of Forestry, Northwest A&F University, 3 Taicheng Road, Yangling 712100, China; 2 VTT Technical Research Centre of Finland, PO Box 1000, FIN-02044 Espoo, Finland; 3 Department of Food and Environmental Sciences, University of Helsinki, PO Box 27, FIN-00014 Helsinki, Finland

## Abstract

**Background:**

Due to the complexity of lignocellulosic materials, a complete enzymatic hydrolysis into fermentable sugars requires a variety of cellulolytic and xylanolytic enzymes. Addition of xylanases has been shown to significantly improve the performance of cellulases and to increase cellulose hydrolysis by solubilizing xylans in lignocellulosic materials. The goal of this work was to investigate the effect of acetyl xylan esterase (AXE) originating from *Trichoderma reesei *on xylan solubilization and enzymatic hydrolysis of cellulose.

**Results:**

The solubilization of xylan in pretreated wheat straw and giant reed (*Arundo donax*) by xylanolytic enzymes and the impact of the sequential or simultaneous solubilization of xylan on the hydrolysis of cellulose by purified enzymes were investigated. The results showed that the removal of acetyl groups in xylan by AXE increased the accessibility of xylan to xylanase and improved the hydrolysis of xylan in pretreated wheat straw and giant reed. Solubilization of xylan led to an increased accessibility of cellulose to cellulases and thereby increased the hydrolysis extent of cellulose. A clear synergistic effect between cellulases and xylanolytic enzymes was observed. The highest hydrolysis yield of cellulose was obtained with a simultaneous use of cellulases, xylanase and AXE, indicating the presence of acetylated xylan within the cellulose matrix. Acetylated xylobiose and acetylated xylotriose were produced from xylan without AXE, as confirmed by atmospheric pressure matrix-assisted laser desorption/ionization ion trap mass spectrometry.

**Conclusions:**

The results in this paper demonstrate that supplementation of xylanase with AXE enhances the solubilization of xylan to some extent and, consequently, increases the subsequent hydrolysis of cellulose. The highest hydrolysis yield was, however, obtained by simultaneous hydrolysis of xylan and cellulose, indicating a layered structure of cellulose and xylan chains in the cell wall substrate. AXE has an important role in the hydrolysis of lignocellulosic materials containing acetylated xylan.

## Background

Plant cell walls consist of three major polymers: cellulose, hemicelluloses and lignin. Cellulose, the most abundant constituent of the plant cell wall, is a homopolysaccharide composed entirely of D-glucose linked together by β-1,4-glucosidic bonds. Xylans, the main hemicelluloses in hardwoods and annual plants, consist of a linear backbone of β-(1→4)-D-xylopyranosyl residues, substituted by α-L-arabinofuranosyl units in the positions of 2-*O *and/or 3-*O*, by 4-*O*-methyl-glucopyranosyl uronic acid in the position of 2-*O*, and/or by acetyl groups in the positions of 2-*O *and/or 3-*O *[1]. Furthermore, some of the arabinofuranosyl units may be esterified with ferulic or *p*-coumaric acids [2].

Complete hydrolysis of lignocellulosic materials to monosaccharides for fermentation to fuels or chemicals can be accomplished by acid hydrolysis, but enzymatic hydrolysis is preferred due to minimization of the formation of byproducts that inhibit the microbial conversion. Due to the complexity of lignocellulosic materials, a complete enzymatic hydrolysis into fermentable monosaccharides requires a variety of cellulolytic and xylanolytic enzymes. Efficient cellulose hydrolysis requires the cooperative action of endoglucanases (E.C. 3.2.1.4), which hydrolyze the cellulose polymer internally, exposing reducing and non-reducing ends, and exoglucanases or cellobiohydrolases (E.C. 3.2.1.91), which act on the reducing or non-reducing ends, releasing mainly cellobiose. The cellulose hydrolysis process is finalized through the action of β-glucosidase (E.C. 3.2.1.21), which cleaves cello-oligosaccharides into two molecules of glucose [3,4]. Given the diversity of xylan structures, a complete hydrolysis of xylan involves the synergistic action of main chain degrading enzymes, including endo-β-1,4-xylanases (EC 3.2.1.8) and β-D-xylosidases (EC 3.2.1.37), and side group cleaving enzymes, including α-L-arabinofuranosidases (EC 3.2.1.55), α-glucuronidases (EC 3.2.1.139), acetyl xylan esterases (AXEs) (EC 3.1.1.72), and feruloyl esterases (EC 3.1.1.73).

In lignocellulosic matrices, xylan is closely associated with the cellulose fibrils, as well as lignin, and does to some extent cover the fiber surfaces, thereby limiting the access of cellulases to the cellulose surface [5]. It has been reported that enzymatic removal of xylan enhances cellulose hydrolysis by removing xylan covering or entrapping cellulose [6,7]. The addition of xylanases (XYLs) has been shown to significantly improve the performance of cellulases and to increase the cellulose conversion of many lignocellulosic materials [8-11]. Thus, the solubilization of xylan in lignocellulosic materials plays an important role in efficient enzymatic hydrolysis.

Hydrolysis of xylan can be enhanced by the removal of xylan side groups; it has been reported that the hydrolysis of isolated hardwood xylans by XYLs was restricted by increasing the degree of acetylation of the xylans [12]. Chemical deacetylation of xylans of aspen wood and wheat straw increased the enzymatic solubilization of xylans and consequently enhanced cellulose accessibility [13]. The improvement of xylan hydrolysis by the concerted action of endoxylanase and AXE has been observed and resulting in further synergistic improvements in cellulose hydrolysis [14]. A relatively linear relationship was observed between the removal of acetyl groups and the release of xylose in the hydrolysis of different corn stover substrates with both endoxylanase and AXE [15]. After pretreatments using hydrothermal or steam explosion treatment of annual plants, some of the substituents, such as acetyl residues, may remain in xylans and hinder the action of XYLs during the enzymatic hydrolysis. In addition, the presence of lignin-carbohydrate complexes between arabinose residues and ferulic or *p*-coumaric acid formed through etherification or esterification has been suggested. Synergism between α-L-arabinofuranosidases and XYLs in the hydrolysis of xylan and agricultural residues has also been reported by Raweesri *et al*. [16]. α-L-arabinofuranosidases from *Aspergillus niger *and endoxylanase from *A. nidulans *synergistically enhanced the hydrolysis of steam exploded wheat straw by cellulases [17]. In order to further elucidate the enzymatic hydrolysis of complex lignocellulosic materials, it is necessary to investigate the solubilization of xylan by xylan-acting enzymes and the consequential effect on cellulose hydrolysis.

In this work, the synergistic action of xylanolytic enzymes in the solubilization of xylan and the subsequent enhancement of cellulose hydrolysis was carried out sequentially or simultaneously with pure cellulases on pretreated wheat straw and giant reed (*Arundo donax*). The role of xylan solubilization with xylanolytic enzymes was investigated by analyzing the carbohydrates, including monosaccharides, disaccharides and xylo-oligosaccharides, released from the substrates.

## Results and discussion

### Solubilization of xylan by xylanolytic enzymes

The impact of various xylanolytic enzymes in the solubilization of xylan from the pretreated wheat straw and giant reed was first investigated (Table 1). After 24 h of hydrolysis by XYL alone, the degree of xylan hydrolysis in the pretreated wheat straw and giant reed by XYL reached 9.8% and 13.2%, respectively. The values in Table 1 include also corresponding acetylated xylo-oligosaccharides, which are saponified in a high performance anion exchange chromatography coupled with pulsed amperometric detection (HPAEC-PAD) analysis. The results clearly showed that the solubilization of xylan in the pretreated substrates by XYL alone was low. Low solubilization (less than 5%) of xylan has also been reported when birch wood was hydrolyzed with a high XYL dosage (5,000 nkat/g substrate) for 24 h [18]. A maximum degree of 11% of xylan hydrolysis in wheat straw was obtained by a very high dosage of pre-adsorbed XYL (0.3 mg protein/g substrate) within 5 h [19]. The reported and present data consistently show that XYL alone was able to remove only a low part of xylan from lignocellulosic materials. The main product in hydrolysates from the pretreated wheat straw and giant reed by XYL was xylobiose, corresponding to 7.2% of wheat straw and 7.5% of giant reed xylans, respectively (Table 1). It has been reported that oligomers from xylobiose to xylopentaose were released from isolated xylans by XYL from *Thermoascus aurantiacus *during initial hydrolysis and xylose and xylobiose were the main products after prolonged hydrolysis [20]. The results here were thus in accordance with those reported results. There was no acetic acid released from the substrates by XYL alone.

**Table 1:** Carbohydrates released from pretreated wheat straw and giant reed by xylan-acting enzymes after 24 h hydrolysis in 50 mM sodium citrate buffer at pH 5.0 and 45°C

Substrate	Enzyme preparation	Xylose	Xylobiose	Xylotriose	Xylotetraose	Total
Wheat straw	XYL	1.6	7.2	1.0	bdl	9.8
	XYL+ARA	1.6	8.0	1.2	bdl	10.8
	XYL+AXE	2.2	9.1	bdl	bdl	11.3
Giant reed	XYL	1.7	7.5	2.3	1.7	13.2
	XYL+ARA	1.6	7.3	2.3	1.8	13.0
	XYL+AXE	3.1	10.6	bdl	bdl	13.7

The results are expressed as % of xylan (as xylose) in wheat straw or giant reed. The values of xylobiose, xylotriose and xylotetraose contained both acetylated and non-acetylated xylo-oligosaccharides. ARA: α-L-arabinofuranosidase; AXE: acetyl xylan esterase; bdl: below detection limit; XYL: xylanase.

There was no obvious synergistic effect observed between XYL and α-arabinofuranosidase, most probably due to the low content of the residual L-arabinofuranosyl side groups in the xylans of wheat straw (0.05%) and giant reed (0.3%). The synergy between XYL and arabinofuranosidase in the degradation of arabinoxylan, rich in arabinose substituents, has previously been described by Sørensen *et al*. [21,22]. The hydrolysis degree of xylans in the pretreated substrates reached 11.3% and 13.7%, respectively, when AXE was added to XYL (Table 1). Supplementation of XYL with AXE caused a clear increase in the amount of xylose and xylobiose, whereas no xylotriose or xylotetraose were detected. After the supplementation of XYL with AXE, 1.1% and 2.6% of acetyl groups (of the original xylan in the substrate) were released from wheat straw and giant reed, respectively. The acetyl groups in lignocellulosic materials are known to restrict the action of XYLs and limit the solubilization of xylan in the substrates [13,15,23]. The synergy between esterase and XYLs has been reported previously [24,25]. Chemical or enzymatic removal of acetyl groups has been shown to increase the availability of the xylan backbone to XYLs and improve the hydrolysis [13,15]. In the present work, the addition of AXE enhanced the solubilization of xylan to some extent, which indicated that AXE removed some acetyl groups in xylan and increased the accessibility of xylan to XYL. The AXE from *Trichoderma reesei *has high specificity for acetylated xylan and it has no activity towards acetylated galactoglucomannan or phenolic substituents from wheat straw xylan [26].

### Influence of xylan solubilization on cellulose hydrolysis

**Figure 1:**
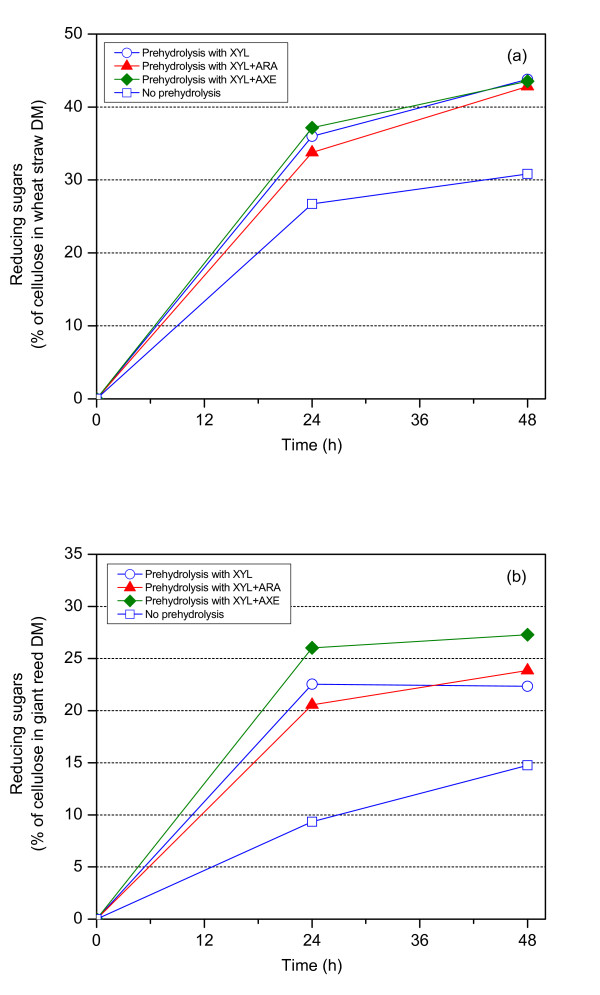
**Hydrolysis of (a) wheat straw and (b) giant reed by a mixture of cellulolytic enzymes (CEL) after 24 h of prehydrolysis by different xylanolytic enzymes in 50 mM sodium citrate buffer at pH 5.0 and at 45°C**.

After the hydrolysis of the pretreated wheat straw and giant reed by different xylanolytic enzymes (prehydrolysis), the washed enzymatic hydrolysis residues were hydrolyzed by a mixture of cellulolytic enzymes (CEL), not containing xylanolytic activities, and the released carbohydrates were determined (Figure 1). Previously, the cellulase preparations used have contained cellulases such as endoglucanase I with side activity towards xylan, preventing detailed studies of the solubilization mechanism of xylans versus cellulose. The small differences in the degree of xylan removed by various enzyme combinations during the prehydrolysis were reflected in the hydrolysis of cellulose in wheat straw by CEL (Figure 1a). A relatively higher extent of xylan was solubilized in giant reed (Table 1) in the prehydrolysis by xylanolytic enzymes, which resulted in a more pronounced increase of hydrolysis by CEL. The degree of hydrolysis of giant reed was, however, low, obviously due to a less severe pretreatment. The maximum extent of cellulose hydrolysis was obtained from giant reed after prehydrolysis by XYL with AXE (Figure 1b). The results revealed that xylan solubilization in the prehydrolysis increased the accessibility of cellulose to cellulases and consequently increased the degree of cellulose hydrolysis. The results indicated that xylan solubilization is important for the hydrolysis of xylan-containing lignocellulosic materials, which is in accordance with other published results [10,11,14].

### Simultaneous hydrolysis of xylan and cellulose

**Figure 2:**
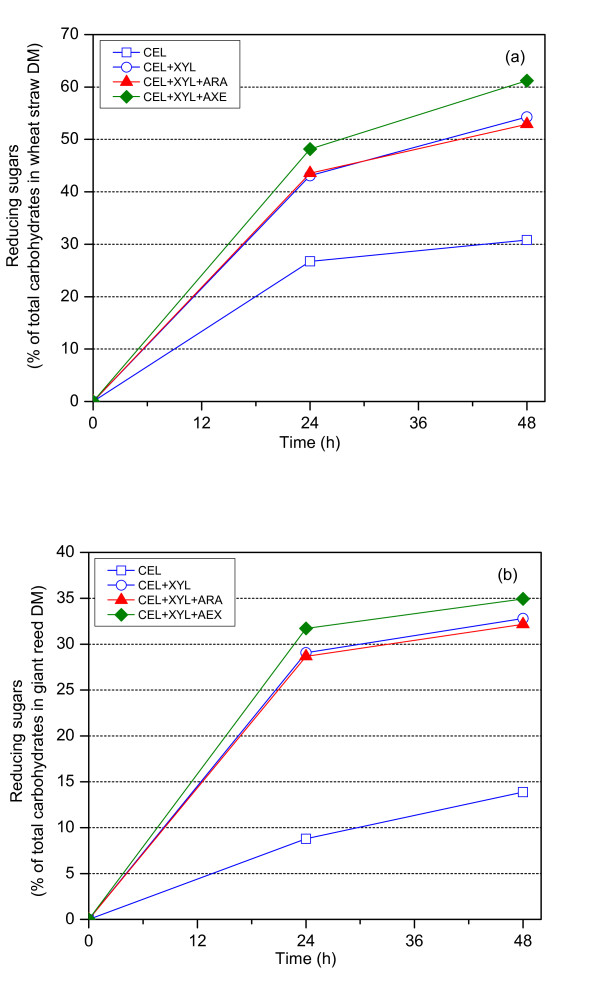
**Hydrolysis of (a) wheat straw and (b) giant reed by combined cellulases and xylanolytic enzymes in 50 mM sodium citrate buffer at pH 5.0 and at 45°C**.

In order to investigate the synergy between different xylanolytic enzymes and cellulases in the hydrolysis of the pretreated wheat straw and giant reed, hydrolysis with combined cellulases and xylanolytic enzymes was carried out (Figure 2 and Table 2). Supplementation of enzymes specific to cellulose hydrolysis (CEL) with xylanolytic enzymes clearly increased the hydrolysis degree of the substrates compared with cellulases alone (Figure 2). With the assistance of xylanolytic enzymes, the hydrolysis yield of cellulose was significantly increased and also more xylan was solubilized (Table 2).

**Table 2:** Carbohydrates released from pretreated wheat straw and giant reed after 48 h hydrolysis by cellulases and xylan-acting enzymes in 50 mM sodium citrate buffer at pH 5.0 and 45°C

Substrate	Enzyme preparation	Glucose	Xylose	Xylobiose	Xylotriose	Cellobiose
Wheat straw	CEL	28.2	bdl^a^	2.9	0.4	0.5
	CEL+XYL	50.4	7.7	34.6	5.7	1.2
	CEL+XYL+ARA	46.6	9.8	29.8	4.6	0.9
	CEL+XYL+AXE	55.6	17.7	46.6	bdl	1.0
Giant reed	CEL	10.8	2.1	2.1	1.1	0.1
	CEL+XYL	26.6	5.5	16.4	5.2	0.1
	CEL+XYL+ARA	26.8	5.9	16.6	5.2	0.1
	CEL+XYL+AXE	28.9	8.7	21.1	bdl	0.1

Glucose and cellobiose are expressed as % of cellulose (as glucose), and xylose, xylobiose and xylotriose are expressed as % of xylan (as xylose) in wheat straw or giant reed. The values of xylobiose, xylotriose and xylotetraose contained both acetylated and non-acetylated xylo-oligosaccharides. ARA: α-L-arabinofuranosidase; AXE: acetyl xylan esterase; bdl: below detection limit; CEL: mixture of cellulose-acting enzymes; XYL: xylanase.

As expected from the results above, there was no obvious enhancement of the simultaneous action of CEL plus XYL on the pretreated wheat straw and giant reed by addition of α-arabinofuranosidase (Figure 2). The highest contents of carbohydrates were released from the pretreated substrates by the combined action of CEL plus XYL plus AXE (Table 2); a consequence of the most extensive xylan solubilization. The amount of acetyl group released from the substrates after supplementation of CEL and XYL with AXE was 4.5% and 3.6% of the original xylan in the pretreated wheat straw and giant reed, respectively. The addition of AXE improved the release of acetyl groups from xylans and subsequently increased the solubilization of xylans, leading to an enhanced action of CEL via improvement of cellulose accessibility and solubilization. The results were in good agreement with reports on the combination of endoxylanase and AXE in increasing the xylan conversion, and consequently the cellulose conversion to glucose by cellulolytic enzymes [14,15].

**Figure 3:**
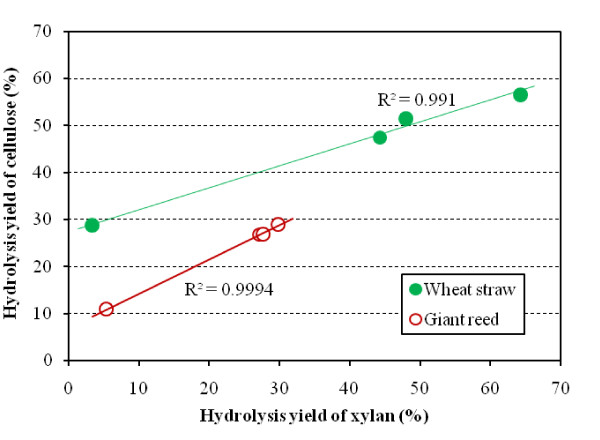
**Relationship of hydrolysis yields between cellulose and xylan in wheat straw (filled circle) and giant reed (open circle) after 48 h hydrolysis by combined cellulases and xylanolytic enzymes in 50 mM sodium citrate buffer at pH 5.0 and at 45°C**. The hydrolysis yield of cellulose was calculated from glucose and cellobiose released from substrates. The hydrolysis yield of xylan was calculated from xylose, xylobiose and xylotriose released from substrates. The calculations were based on monosaccharides in the raw materials.

The degree of hydrolysis of both xylan and cellulose was significantly higher when the enzymes were used simultaneously as compared to the sequential hydrolysis experiments. The synergistic relationship between the hydrolysis yields of xylan and cellulose in the pretreated wheat straw and giant reed is shown in Figure 3. The total hydrolysis yield of xylan includes the sum of xylose, xylobiose and xylotriose and the total yield of cellulose contains glucose and cellobiose. It was found that the hydrolysis yield of cellulose in the substrates was increased nearly linearly with the solubilization of xylan. The results indicated that xylanolytic enzymes improved the access of cellulases to cellulose by solubilizing xylan, presumably coating cellulose fibers, as suggested previously [7,14,15,27]. Obviously, xylan is located throughout the fibrous substrate, and it seems that xylan coats cellulose even on a microfibrillar level. As shown in Figure 3, more xylan had to be hydrolyzed to reach the same level of cellulose hydrolysis in giant reed. Meanwhile, a high cellulose hydrolysis yield could be obtained in wheat straw when the same level of xylan was hydrolyzed. Such a difference in hydrolysis yield was caused by the varying pretreatment conditions and substrate structure, resulting in a higher residual amount of xylan in giant reed. The synergistic effect of xylanolytic enzymes with cellulases was evaluated by a synergy factor, which was calculated from the ratio of glucose released by the combination of cellulases and xylanolytic enzymes and the total amount of glucose released by enzymes alone, as described previously [28]. The synergy factors were increased from 1.79 and 2.46 to 1.97 and 2.67, respectively, in wheat straw and giant reed when AXE was added to CEL plus XYL. A higher synergy factor was obtained in giant reed due to the higher xylose content (7.7%) as compared with wheat straw (3.6%). These results revealed that supplementation of CEL plus XYL with AXE increased the synergistic effect of xylanolytic enzymes with cellulases, and the synergy was more pronounced in lignocellulosic materials containing high xylan content.

The amount of xylose, xylobiose and xylotriose released by the combination of cellulases and xylanolytic enzymes was higher than released by corresponding xylanolytic enzymes alone (Tables 1 and 2). Obviously, hydrolysis with xylanolytic enzymes with the addition of CEL increased the solubilization of xylan in the substrates. The highest hydrolysis degrees of xylans in the pretreated wheat straw and giant reed were 64.3% and 29.8% (compared with 11.3% and 13.7%, respectively, in the absence of cellulose hydrolysis). The xylans in the substrates were not fully solubilized due to structural limitations caused by associations of hemicelluloses, cellulose and lignin in the plant cell wall [5]. The degree of cellulose hydrolysis by the combination of CEL plus XYL with AXE followed the same trend, thus being significantly higher (55.6%) in wheat straw and in giant reed (28.9%) (Table 2). The final degree of cellulose hydrolysis may also have been limited by the number of cellulases chosen in this work, excluding some of the endoglucanases and other essential minor cellulolytic enzymes.

### Action of AXE in enzymatic hydrolysis

**Figure 4:**
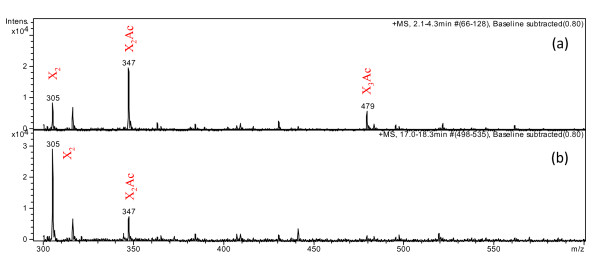
**Atmospheric pressure matrix assisted laser desorption/ionization ion trap mass spectrometer mass spectra showing ion peaks (sodium adducts) of hydrolysates from giant reed after 48 h hydrolysis by (a) cellulases and xylanolytic enzymes and (b) cellulases and xylanolytic enzymes with acetyl xylan esterase in 50 mM sodium citrate buffer at pH 5.0 and at 45°C**.

No xylotriose or higher degree of polymerization xylo-oligosaccharides were released when wheat straw and giant reed were hydrolyzed by different enzyme preparations with AXE (Tables 1 and 2). As expected, more xylose and xylobiose was always released from the substrates by XYL with AXE and CEL plus XYL with AXE, as compared with by XYL and CEL plus XYL alone, respectively. During the hydrolysis with esterase and xylanase, the removal of acetyl groups increased the sites for xylanase hydrolysis and shorter xylo-oligosaccharides were formed, as observed earlier [29]. Shorter oligosaccharides were released from xylan by XYL and AXE compared with by XYL alone. Previously, the XYL from *T. aurantiacus *has been shown to have a typical hydrolytic pattern of glycoside hydrolase family 10 enzymes on birchwood glucuronoxylan and oat spelt arabinoxylan [18,30]. The enzyme cleaves the glycosidic linkages in the xylan main chain closer to the substituent. The enzyme was shown to released arabinoxylobiose as the shortest arabinoxylo-oligosaccharide from oat spelt arabinoxylan and aldotetrauronic acid as shortest acid xylo-oligosaccharide from birchwood glucuronoxylan [20]. Xylobiose in hydrolysates from wheat straw and xylobiose and xylotriose from giant reed were accumulated to some extent due to the acetylation as confirmed by mass spectrometry analysis. Oligosaccharides in hydrolysates from giant reed by CEL plus XYL (Figure 4a) and CEL plus XYL with AXE (Figure 4b) were analyzed by atmospheric pressure matrix assisted laser desorption/ionization ion trap mass spectrometer (AP-MALDI-ITMS). The major ion in Figure 4a was observed at *m/z *347, which was the sodium adduct [M+Na]^+ ^of acetyl xylobiose. The ions at *m/z *305 and 479 correspond to [M+Na]^+ ^of xylobiose and acetyl xylotriose. In Figure 4b, the major ion was observed at *m/z *305 corresponding to [M+Na]^+ ^of xylobiose. Interestingly, the ion of sodium adduct [M+Na]^+ ^of acetyl xylobiose was also observed. Although xylotriose was observed in HPAEC-PAD of the hydrolysate with CEL plus XYL, the sodium adduct of xylotriose was not observed in the AP-MALDI-ITMS spectra. This confirms that xylotriose quantified by the HPAEC-PAD analysis was completely monoacetylated. In this work, xylobiose in hydrolysates from wheat straw and xylobiose and xylotriose from giant reed were accumulated to some extent due to the acetylation as confirmed by mass spectrometry analysis. The AXE from *T. reesei *is highly active on polymeric xylan [26,29]. However, it has a strong preference for deacetylation of position 2 in the xylopyranoses [31]. Thus, the residual acetylated xylobiose detected might carry acetylation on position 3 or even 4, as acetyl groups are known to be prone to migration [31]. It has been reported previously that both *T. reesei *AXE (carbohydrate esterase family 6) and acetyl esterase (carbohydrate esterase family 16), with specificity on positions 3 and 4, were needed for complete deacetylation of xylan [26,31]. According to the CAZy database, *T. reesei *(*Hypocrea jecorina*) is reported to have also another carbohydrate esterase family AXE (AXE2) (http://www.cazy.org). Efficient deacetylation is important for increasing the hydrolysis yield of xylan and cellulose, as well as for producing non-acetylated hydrolysis products for further conversions.

## Conclusions

Solubilization of xylan by xylanolytic enzymes before the addition of pure cellulases clearly increased the hydrolysis yield of cellulose in pretreated wheat straw and giant reed. Supplementation of XYL with AXE enhanced the solubilization of xylan only slightly and had a minor effect on the subsequent hydrolysis of cellulose. The hydrolysis of xylan, however, was dramatically increased along with simultaneous hydrolysis of cellulose, indicating a layered structure of cellulose and xylan chains occurring alternately in the cell wall substrate. Also, the total hydrolysis yield was significantly increased by the simultaneous action of xylanases and cellulases. A linear relationship between the hydrolysis yields of xylan and cellulose was thus obtained, which showed clear synergism between CEL and xylanolytic enzymes in the hydrolysis of wheat straw and giant reed. The presence of AXE clearly increased the simultaneous solubilization and hydrolysis of xylan and cellulose. The XYL used produced xylobiose as the main hydrolysis product. No xylotriose was detected in the hydrolysates from the substrates after the addition of AXE, and more xylose and xylobiose was detected. The results indicated that AXE could remove acetyl group from xylan in the substrates and released xylan fragments, creating new sites for XYL action and thus leading to the formation of shorter carbohydrates and increased xylan solubilization. The data in this work supports the finding that AXE has an important role in the hydrolysis of pretreated wheat straw and giant reed.

## Methods

### Chemicals

The monosaccharides, D-xylose and D-glucose were purchased from Merck (Darmstadt, Germany) and 1,4-β-D-xylobiose, 1,4-β-D-xylotriose, 1,4-β-D-xylotetraose and cellobiose were from Megazyme (Bray, Ireland). All other chemicals used were analytical grade.

### Materials

Hydrothermally pretreated wheat straw was a kind gift from Inbicon (Fredericia, Denmark). The carbohydrate composition of the wheat straw as monosaccharides was glucose 65.4%, xylose 3.6% and arabinose 0.05% of dry matter (DM). The pretreated giant reed *(A. donax) *was kindly provided by Chemtex (Tortona, Italy). The carbohydrate composition of the giant reed as monosaccharides was glucose 49.8%, xylose 7.7% and arabinose 0.3% of DM. The amounts of acetyl groups were 0.12% and 0.44% of DM in the pretreated wheat straw and giant reed, respectively.

### Enzymes

Cellobiohydrolases CBH I (Cel 7A), CBH II (Cel 6A), and endoglucanase EG II (Cel 5A) from *T. reesei *were purified as described by Suurnäkki *et al*. [32]. *A. niger *β-glucosidase (βG) (Cel 3A) was purified from Novozyme 188 (Novozymes A/S, Bagsvaerd, Denmark) according to Sipos *et al*. [33]. XYL (family 10) from *Thermoascus aurantiacus *was a kind gift from ROAL and was partially purified from the preparation containing XYL, heterologously produced by the genetically modified *T. reesei *strain [34]. AXE from *T. reesei *was purified as described by Sundberg and Poutanen [35]. The α-L-arabinofuranosidase was from Novozymes. XYL activity was assayed using birchwood glucuronoxylan as a substrate in 50 mM sodium citrate buffer (pH 5.0) according to the method of Bailey *et al*. [36]. AXE activity was assayed using *p*-nitrophenyl acetate as a substrate [35] and α-L-arabinofuranosidase activity using *p*-nitrophenyl-α-L-arabinofuranoside as a substrate [12]. Protein was quantified by the Lowry method, using BSA (St. Louis, MO, Sigma Chemical Co., USA) as standard [37].

### Enzymatic hydrolysis

In order to investigate the solubilization of xylan in the pretreated wheat straw and giant reed by different xylanolytic enzyme mixtures and examine the influence of xylan solubilization on further hydrolysis of substrates by cellulases, the enzymatic hydrolysis experiments were carried out in two steps. In the prehydrolysis step, the hydrolysis of the pretreated wheat straw and giant reed was carried out by the different xylanolytic enzyme mixtures in a working volume of 2.5 mL in 50 mM sodium citrate buffer (pH 5.0) containing 0.02% NaN_3 _at 45°C. All hydrolysis experiments were carried out in duplicates. The DM content of the substrate was 2%. XYL was dosed at 5 000 nkat/g substrate (0.36 mg protein/g substrate), and AXE and α-L-arabinofuranosidase were both dosed at 500 nkat/g substrate (0.17 mg protein/g substrate for α-L-arabinofuranosidase). The dosages of different enzymes used were always the same throughout the experiments unless otherwise noted. After 24 h of hydrolysis, all samples were withdrawn and boiled for 10 min to stop the enzymatic hydrolysis. After cooling, the samples were washed three times with Milli-Q-water (Milli-Q-plus, Millipore, Billerica, MA, USA) and the supernatants were combined for analysis. In the second step, the hydrolysis residues were further hydrolyzed by the CEL containing CBHI 6.5 mg, CBHII 1.5 mg, EGII 2.0 mg and βG 500 nkat/g substrate in the same conditions as described previously. Samples were withdrawn at 24 h and 48 h and boiled for 10 min to stop the enzymatic hydrolysis.

The simultaneous hydrolysis of the pretreated wheat straw and giant reed by cellulolytic enzymes (CEL) and different xylanolytic enzymes was carried out as described above. The DM content of substrate was 2%. CEL (CBHI plus CBHII plus EGII plus βG) was dosed and loaded in all samples as described earlier and XYL, AXE and α-L-arabinofuranosidase were added to examine the synergistic enhancement between cellulases and different xylanolytic enzymes. Samples were withdrawn at 24 h and 48 h and boiled for 10 min to stop the enzymatic hydrolysis. All samples were centrifuged and the supernatants were analyzed for reducing sugars. Two replicate tests were carried out in all hydrolysis experiments and average values were presented. The reducing sugar concentrations showed a relative standard error of less than 4%. Monosaccharides, disaccharides and oligosaccharides were analyzed by HPAEC-PAD. The relative standard errors were determined for the standards and were shown to be consistently below 1%.

### Analysis of carbohydrates

The amount of reducing sugars liberated was determined using the dinitrosalicylic acid method with xylose or glucose as standard [38]. Monosaccharides in the hydrolysates were analyzed using HPAEC-PAD system equipped with Waters 2707 autosampler, Waters 515 HPLC pumps and Waters 2465 pulsed amperometric detector using Empower 2 software for instrument control and data analysis (Waters Corporation, Milford, MA, USA). An analytical CarboPac PA-1 column (4.0 mm ID × 250 mm) in combination with a CarboPac PA-1 guard column (4.0 mm × 50 mm) (Dionex, Sunnyvale, USA) was used. For the analysis of monosaccharides, the eluents for gradient analysis were H_2_O and 0.2 M NaOH with a total flow rate of 1 mL/min with post column addition of 300 mM NaOH at a flow rate of 0.3 mL/min. D-glucose, D-xylose, and D-arabinose (Merck, Darmstadt, Germany) were used as external standards [39].

The analytical CarboPac PA-100 column (250 mm × 4 mm) and the guard column PA-100 (25 mm × 3 mm) (Dionex) were used for disaccharide and oligosaccharide analysis. The eluents for gradient analysis were 100 mM NaOH and 100 mM NaOH/1 M NaOAc at a flow rate of 1 mL/minute as described by Rantanen *et al*. [40]. It should be observed that the acetyl substituents in the oligosaccharides were removed during the HPAEC-PAD analysis due to the alkaline eluent. 1,4-β-D-cellobiose, 1,4-β-D-xylobiose, 1,4-β-D-xylotriose and 1,4-β-D-xylotetraose (Megazyme, Bray, Ireland) were used as external standards.

### Analysis of acetyl groups

The content of acetyl groups of the substrates was analyzed using the Megazyme Acetic Assay Kit (K-ACET) after acid hydrolysis of the samples.

### AP-MALDI-ITMS analysis

The hydrolysates of giant reed were desalted and concentrated using Hypersep Hypercarb Porous Graphitized Carbon columns (Thermo Scientific, Waltham, USA), according to the established protocols by Packer *et al*. [41]. The oligosaccharides were eluted from the column by 50% (v/v) CH_3_CN in 0.05% (v/v) trifluoroacetic acid. Eluted oligosaccharides were dried and dissolved in 30 μL of Mili-Q-water. The 2,5-dihydroxybenzoic acid (DHB) solution (10 mg/mL) was employed as a matrix for AP-MALDI-ITMS measurement. The 10 mg DHB was dissolved in 1 mL CH_3_CN/Milli-Q-water (3:7, v/v). The oligosaccharides were crystallized on a target plate by mixing 1 μL of the sample with 1 μL of the DHB solution, followed by drying under a constant stream of warm air. The AP-MALDI-ITMS was operated according to the protocols described by Chong *et al*. [42].

## List of abbreviations

AP-MALDI-ITMS: atmospheric pressure matrix assisted laser desorption/ionization ion trap mass spectrometer; AXE: acetyl xylan esterase; βG; β-glucosidase; BSA: bovine serum albumin; CBH: cellobiohydrolases; CEL: mixture of cellulose-acting enzymes; DHB: 2,5-dihydroxybenzoic acid; DM: dry matter; EG: endoglucanase; HPAEC-PAD: high-performance anion exchange chromatography coupled with pulsed amperometric detection; XYL: xylanase.

## Competing interests

The authors declare that they have no competing interests.

## Authors' contributions

JZ carried out the experimental work, analyzed the results and drafted the manuscript. MS reviewed the paper. LV conceived, designed and coordinated the overall study and helped to analyze the results and finalize the paper. MT discussed the analysis of the results and commented on the text. All authors read and approved the final manuscript.
